# Risk Evaluation of Construction Workers’ Exposure to Silica Dust and the Possible Lung Function Impairments

**Published:** 2017-06

**Authors:** Elahe Tavakol, Mansour Azari, Rezvan Zendehdel, Sousan Salehpour, Soheila Khodakrim, Saeed Nikoo, Behzad Saranjam

**Affiliations:** 1 College of Public Health, Shahid Beheshti University of Medical Sciences, Tehran Iran,; 2 Safety Promotion and Injuries Prevention Research Center, College of Public Health, Shahid Beheshti University of Medical Sciences, Tehran, Iran,; 3 School of Public Health, School of Paramedical Science, Shahid Beheshti University of Medical Sciences, Tehran, Iran,; 4 Ministry of Roads & Urban Development, Islamic Republic of Iran.

**Keywords:** Construction, Crystalline silica, PNOS, Respiratory system

## Abstract

**Background:**

Aerosols generated during construction activities are an integral part of building operations. Considering the nature of materials used in construction activities, respirable dust contains crystalline silica and particulates not otherwise specified (PNOS). Due to lack of data regarding the occupational health status of Iranian construction workers, the objective of this study was to evaluate occupational exposure to silica and to examine their respiratory health status.

**Materials and Methods:**

In this cross sectional study, 85 construction workers and 40 controls (without active exposure to construction dust) were studied. The workers’ exposure to PNOS and silica aerosols was monitored by the NIOSH method No.0600 and a new Fourier transform infrared spectroscopy (FTIR)-based method, respectively. All subjects were also monitored for lung function parameters, such as forced expiratory volume/forced vital capacity (FEV_1_/FVC), peak expiratory flow rate (PEFR), forced expiratory flow (FEF_25–75_), FVC, and FEV_1_.

**Results:**

The mean exposure of workers to respirable PNOS and silica was 9.8 (0.35) and 0.13 (0.019) mg/m^3^, respectively. The groups of construction workers showed significant differences in exposure to PNOS (*P*< 0.001) and silica (*P*= 0.007). The mean pulmonary function parameters, including FEV_1_% and FVC%, were significantly lower among construction workers, compared to the control group (*P*< 0.001 and *P*= 0.009, respectively). The pulmonary status of 51.8% of construction workers showed moderate restriction, while 4.70% exhibited obstruction.

**Conclusion:**

Considering the construction workers’ excessive exposure to PNOS and silica, besides depressed lung function parameters, they can be classified as a high-risk group for respiratory diseases.

## INTRODUCTION

Construction activities reportedly generate dusts, which can be a risk factor for workers’ respiratory dysfunctions (1–4). Lung function impairment is the most common respiratory problem among workers exposed to dusts (5). Construction workers are especially exposed to high concentrations of dusts in closed spaces and breathe high levels of crystalline silica (6–9). The International Agency for Research on Cancer (IARC), based on sufficient evidence of carcinogenicity, has classified crystalline silica as a group I carcinogen and a definite human carcinogen (9, 10). The American Conference of Governmental Industrial Hygienist (ACGIH) has also classified silica in group A2 as a probable carcinogen (11). Due to the carcinogenicity of crystalline silica, ACGIH reduced the threshold limit value (TLV) of crystalline silica from 0.1 mg/m^3^ in 1986 to 0.025 mg/m^3^ in 2006 (12).

Silicosis is recognized as a restrictive pulmonary disease. It has been described as the most prevalent respiratory disease since 1968 due to silica dust exposure and is now considered a global problem (13–15). Since 1995, the World Health Organization (WHO) in conjunction with the International Labor Office (ILO) has managed a Global Program for the Elimination of Silicosis since 1995, while the National Institute for Occupational Safety and Health (NIOSH) in the United States initiated a program in 2005, known as Elimination of Silicosis in the Americas [16].

Considering the nature of materials used in the construction industry, dust may contain significant amounts of crystalline silica (16, 17). There is substantial epidemiological evidence in relation to occupational exposure to respirable general dusts, which contain less than 1% silica and are classified as particulate not otherwise specified (PNOS), as well as respirable crystalline silica, associated with the development of various diseases, such as silicosis, lung cancer, tuberculosis, and pulmonary obstructive disease (15, 18–21).

Based on the NIOSH report, the highest rate of mortality from silicosis was related to construction activities among all other industries during 1990–1999 (22).

Reduced lung function parameters, such as forced expiratory volume/forced vital capacity (FEV_1_/FVC), peak expiratory flow rate (PEFR), forced expiratory flow (FEF_25–75_), FVC, and FEV_1_ due to cumulative exposure to respirable PNOS and silica dust, along with airway obstruction, have been reported in several construction task groups, working with materials such as concrete, ceramic, and bricks (23–26). Respiratory problems, associated with changes in chest radiographs and pulmonary function, were also reported among construction workers (27, 28).

Spirometric parameters can be used to distinguish obstructive and restrictive lung status in adults. According to the criteria by Ford et al. and Mannino et al., obstructive and restrictive pulmonary status is defined as follows: severe obstructive impairment (FEV_1_/FVC< 0.70; FEV_1_< 50% predicted), moderate obstructive impairment (FEV_1_/FVC< 0.70; FEV_1_ 50% to < 80% predicted), mild obstructive impairment (FEV_1_/FVC < 0.70; FEV_1_ ≥ 80% predicted), and restrictive impairment (FEV_1_/FVC≥ 0.70; FVC< 80% predicted) (29, 30).

Various methods for the analysis of crystalline silica have been proposed by the scientific and executive organizations. These methods include X-ray spectrometry (XRD) by OSHA method No. 142 (31), infrared spectrophotometry by NIOSH method No. 7602 (32), and visible spectrophotometry by NIOSH method No. 7601 (33). Fourier transform infrared spectroscopy (FTIR), due to the application of a Fourier algorithm, is more accurate for the recognition of crystalline silica molecular fingerprint in comparison with infrared spectrophotometry (34).

Construction workers reportedly experience greater exposure to respirable crystalline silica and PNOS compared to the occupational exposure limit, and several authorities have recommended further research for better monitoring and control of construction workers (35, 36). According to the International Labor Organization (ILO), silicosis, as a preventable but incurable disease, requires awareness of the quality and quantity of respirable crystalline silica for devising proper control measures (37, 38). Considering the high occupational exposure of construction workers to airborne dusts and absence of relevant studies in Iran, the aim of this study was to evaluate workers’ exposure to respirable PNOS and crystalline silica and to examine their respiratory lung function status.

## MATERIALS AND METHODS

Eighty-five workers from a major construction company, along with 40 control workers without active exposure to respirable crystalline silica and PNOS were randomly selected, according to the statistical calculations related to pulmonary function from a previous study, monitoring a group of construction workers (26). All workers were from the same socioeconomic class, nonsmokers, and healthy with at least 1 year of work history, without any respiratory diseases or prescription drug use. Iranian construction workers were from 5 occupational task groups, including supervisors of construction activities, cleaning crew, and cement, masonry, batching, and concrete workers. The control workers, without active exposure to dust, were recruited from the security personnel of the same site.

In this study, the workers’ personal exposure to respirable PNOS was determined according to the NIOSH method No. 0600 (33). In this method, personal monitoring was performed with an SKC personal sampling pump (model 224-44MTX), which was connected to a nylon cyclone and calibrated by a calibrated rotameter at the flow rate of 1.7 L/min for 4 hours, using dried mixed cellulose ester (MCE) filters. After sampling and drying processes, the filters were weighed with a Sartorius analytical balance at 0.01-mg resolution.

For determining the workers’ personal exposure to respirable crystalline silica dust, a new FTIR-based method by Virji et al. was applied (34). In this method, sampling for respirable dust was carried out as described earlier. To each filter, 200 mg of potassium bromide was added in a crucible dish and subsequently burned in an electric furnace for 4 hours at 600OC. After cooling down, each sample was grinded, homogenized by a mortar, and pressed into a 13-mm tablet, using a press machine at 20-MPa pressure.

The prepared tablets were scanned using an FTIR spectrometer (model WQF-510A) at the wavelength range of 400–4000 cm^−1^. Crystalline silica in the prepared tablets was determined in the range of 710–825 cm^−1^. In order to examine the accuracy of FTIR analysis, 13 bulk samples were analyzed by the FTIR-based method of this study, as well as a reference method combining X-ray fluorescence (XRF) and X-ray diffraction (XRD) analyses (39). For this purpose, an XRD device (PW1800, Philips Co.) was used for the qualitative detection of crystalline silica, and an XRF device (PW1480, Philips Co.) was applied for quantitative detection.

Pulmonary function tests were performed using a spirometer (model 3000, Bionet Cardio Touch) in the exposed and control groups. The characteristics (height, weight, and age) of the exposed and control groups were recorded. The subjects were asked to stand comfortably in front of the spirometer and then inhale and exhale. The pulmonary status was described as restrictive or obstructive, and the spirometric results were interpreted according to the American Thoracic Society (ATS) guidelines (40–42).

Mann-Whitney, ANOVA, and Kruskal-Wallis tests were used for statistical analysis, and quantitative data were reported as mean (standard deviation). Moreover, agreement of 2 sets of continuous data from 1 set of samples (13 samples) was analyzed by 2 different methods (FTIR and combination of XRD and XRF) and examined by the Bland-Altman plot and intraclass correlation (ICC) index.

## RESULTS

The mean age and work experience were 32.32 and 10.8 years in the exposed group and 32.09 and 9.78 years in the control group, respectively. There was no significant difference in terms of age and work experience between the exposed and control groups (*P*> 0.05). Agreement of 2 datasets from 1 set of silica samples (13 samples) was analyzed by 2 different methods (FTIR and combination of XRD and XRF) and examined by the Bland-Altman plot. As the differences were within ±0.2 SD from the average of differences, the agreement of 2 datasets was established (ICC, 0.993; *P*< 0.001).

Table 1 and 2 present the results of personal monitoring of PNOS and respirable crystalline silica, along with the pulmonary function status of 5 construction and control groups. Significant differences were found in terms of exposure to PNOS and respirable crystalline silica in different construction groups (*P*< 0.001 and *P*= 0.007, respectively). Significant differences were also observed in the lung function parameters (FEV_1_% and FVC%) of all construction groups and the controls (*P*< 0.001 and *P*= 0.009, respectively; Table 2).

**Table 1.** Construction workers’ exposure to respirable crystalline silica and PNOS as mg/m^3^

**Construction & Control groups**	**Number of personal**	**Mean PNOS (SE)**	**Mean silica (SE)**	**Mean % Silica per sample**
**Supervisor**	17	(0.58) 7.34	(0.05) 0.133	1.81
**Cement**	17	(0.62) 8.20	(0.07) 0.184	2.24
**Masonry**	17	(0.56) 7.33	(0.020) 0.071	0.97
**Batching & concrete**	17	(0.77) 11.38	(0.025) 0.159	1.40
**Cleaning crew**	17	(0.85) 10.53	(0.022) 0.103	0.98
**All construction groups**	85	(0.35) 8.9	(0.019) 0.13	1.46
**Control group**	40	(0.11) 0.43	< LOD^*^	—

* 5.56 μg per sample

**Table 2.** Pulmonary lung function parameters of construction and control groups

**Construction & Control groups**	**%FVC**	**%FEV_1_**	**/FVC %FEV_1_**	**%FEF_25–75_**
**Supervisors**	79.46	86.05	109.01	110.41
**Cement**	79.68	87.22	109.97	108.04
**Masonry**	79.53	84.36	106.31	100.32
**Batching and Concrete**	85.59	80.55	94.59	105.72
**Cleaning Crew**	77.35	85.27	110.43	110.90
**All construction groups**	80.32	84.69	106.06	107.08
**Control group**	85.53	90.84	106.41	107.06

**Figure 1.**
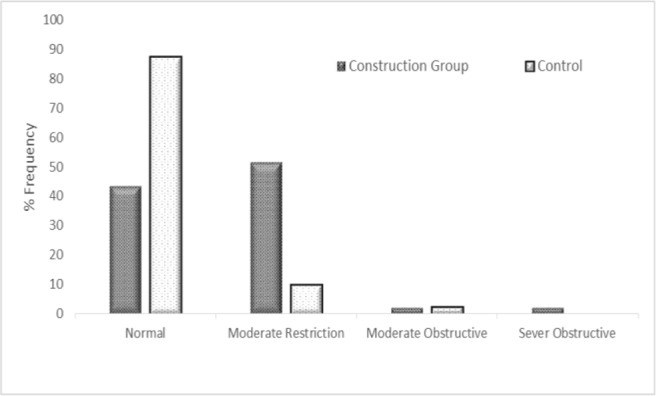
Percentage of pulmonary status of construction and control groups

In addition, significant differences were found between the normal and abnormal pulmonary status of the construction and control groups (*P*< 0.001). The pulmonary status was normal in 43.5% of subjects from the construction groups and 87.5% of subjects from the control group. Also, more than half of the construction workers (51.8%) were diagnosed with moderate restriction, while 4.70% showed the obstructive status (Figure 1).

**Figure 2.**
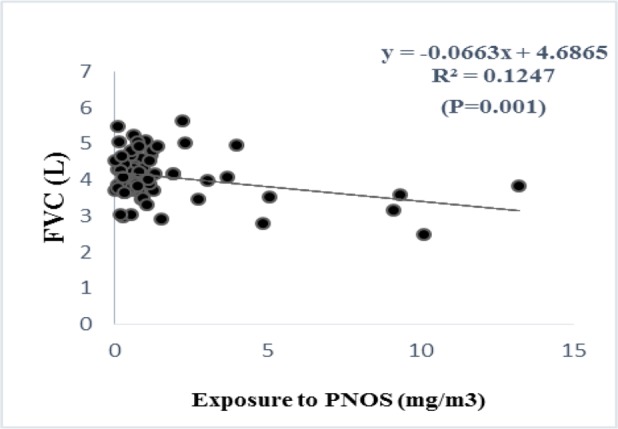
The relationship between exposure to PNOS with FVC

**Figure 3.**
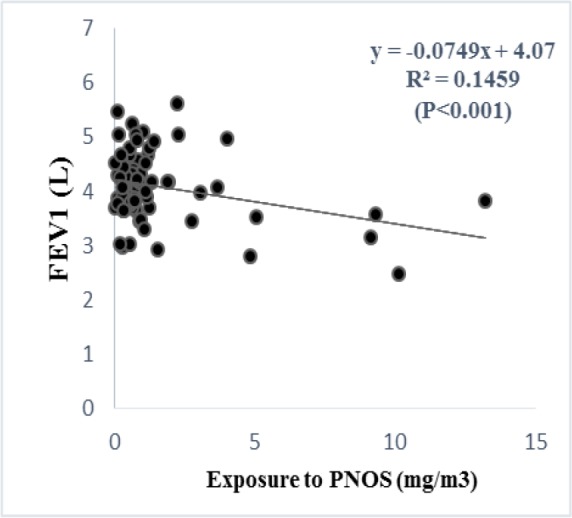
The relationship between exposure to PNOS with FEV_1_

**Figure 4.**
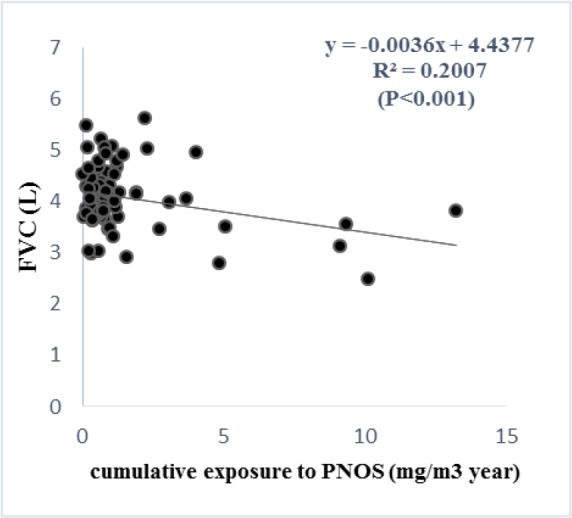
The **relationship** between cumulative cumulative exposure to PNOS with FVC

**Figure 5.**
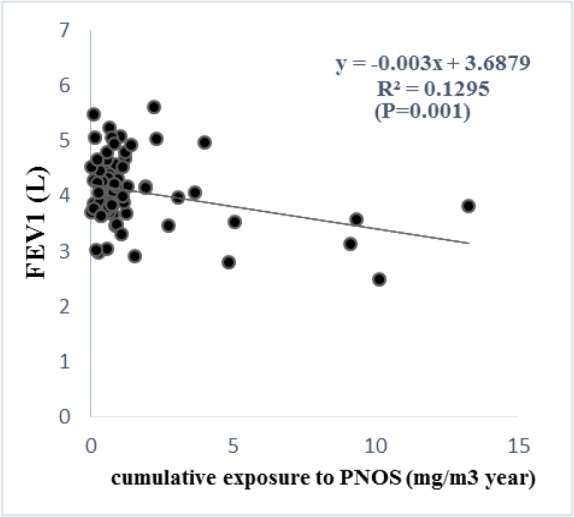
The relationship between exposure to PNOS with FEV_1_

**Figure 6.**
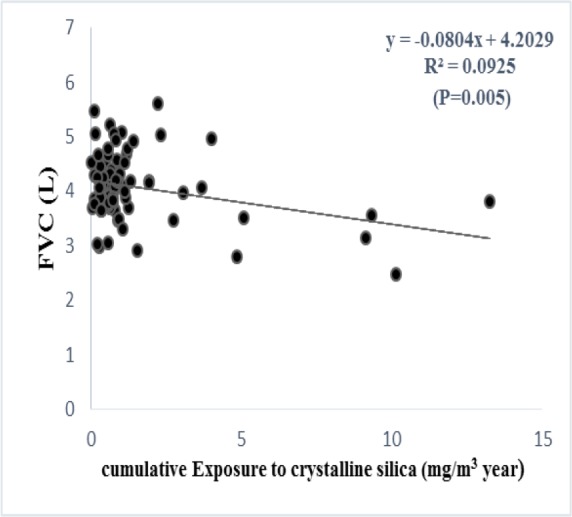
The relationship between the cumulative exposure to crystalline silica dust and FVC

According to the regression analysis, exposure to respirable PNOS and cumulative exposure to PNOS had a significant negative correlation with respiratory parameters, FVC and FEV_1_, respectively (Figures 2–5). A significant negative correlation was also observed between cumulative exposure to respirable crystalline silica dust and the respiratory parameter, FVC (Figure 6).

## DISCUSSION

In this study, there were no significant demographic differences between the construction and control groups. In addition, occupational exposure to PNOS and respirable crystalline silica dust, along with lung function parameters, were evaluated. Among the construction workers, batching and concrete workers showed the highest average exposure to PNOS, which could be due to the type of tasks with continuous exposure in operational processes, such as mixing and transferring sand, gravel, and cement. The cement group had the highest average exposure to respirable crystalline silica dust, which might be attributed to the higher percentage of crystalline silica in Iranian cement, compared to European cement (43).

Almost all workers had higher exposure to crystalline silica than the threshold limit value (TLV) by the Iranian Ministry of Health and ACGIH (0.025 mg/m^3^). On the other hand, all workers had lower exposure to PNOS in comparison with the TLV established by the Iranian Ministry of Health or ACGIH (3 mg/m^3^) (11, 44). However, respirable general dust (PNOS), as described by the authorities, should contain less than 1% crystalline silica (11). According to our findings, all general respirable dusts contained more than 1% silica; therefore, it seems that all construction workers may also experience risky exposure to general respirable dust.

Occupational exposure of construction workers to respirable PNOS and crystalline silica dust in this study was similar to the findings reported by Tjoe-Nij Rappaport and Flanagan among Canadian and American construction workers (45–47). However, occupational exposure of construction workers in our research was higher than exposures in recent studies on Canadian and American construction workers (48, 49). Excessive exposure in this study might be due to lack of engineering control measures and higher percentage of crystalline silica in Iranian cement and PNOS, compared with other countries.

Due to lack of data on the lung function parameters of construction workers in Iran, we examined the workers’ lung function parameters. The mean lung function parameters, including FVC% and FEV_1_%, were significantly lower among exposed construction workers, compared to the control group. Based on the findings, a significant relationship was observed between exposure to PNOS and reduction in pulmonary parameters, such as FVC and FEV_1_.

In this study, reduction in lung function parameters versus the control group was in agreement with the results reported by Johncy, Al-Neaimi, Poornajaf, and Kakooei (50–53). Tjoe-Nij found that obstructive pulmonary status or limitation is associated with exposure to crystalline silica in construction workers (54). However, in this study, more than half of construction workers (51.8%) were diagnosed with moderate pulmonary restriction, and only 4.70% were classified as obstructive.

The observed contrast in the pulmonary status might be attributed to the higher exposure of Iranian workers to crystalline silica with the restrictive status (55). Another reason for this contrast could be the selection of construction workers from a nonsmoker working population, since smoking has been introduced as one of the main causes of chronic obstructive pulmonary disease, resulting in the obstructive pulmonary status (56, 57). However, follow-up studies for the diagnosis of restrictive diseases, total lung capacity measurements, and chest X-ray are recommended for more precise results.

## CONCLUSION

Construction workers’ exposure to respirable PNOS dust and crystalline silica dust exceeded the TLV set by ACGIH. Also, a considerable percentage of construction workers demonstrated a moderately restrictive pulmonary status; therefore, Iranian construction workers definitely require more thorough medical examinations. Considering their occupational exposure to silica dust and spirometric data, this population is at risk, and health-promoting activities, such as use of control measures and health education for encouraging them to remain nonsmokers, are recommended.
